# Male Infertility: Causes and Management at a Tertiary Care Center in India

**DOI:** 10.7759/cureus.45584

**Published:** 2023-09-20

**Authors:** Sumesh Choudhary, Vineet Mishra, Pritti Kumari, Hardik Sheth, Rahnuma Ahmad, Mainul Haque, Santosh Kumar

**Affiliations:** 1 Department of Obstetrics and Gynecology, Smt. G. R. Doshi and Smt. K. M. Mehta Institute of Kidney Diseases and Research Center and Dr. H. L. Trivedi Institute of Transplantation Sciences, Ahmedabad, IND; 2 Department of Obstetrics and Gynecology Genetics, Smt. G. R. Doshi and Smt. K. M. Mehta Institute of Kidney Diseases and Research Center and Dr. H. L. Trivedi Institute of Transplantation Sciences, Ahmedabad, IND; 3 Department of Physiology, Medical College for Women and Hospital, Dhaka, BGD; 4 Department of Research, Karnavati Scientific Research Center (KSRC) Karnavati School of Dentistry, Karnavati University, Gandhinagar, IND; 5 Department of Pharmacology and Therapeutics, National Defence University of Malaysia, Kuala Lumpur, MYS; 6 Department of Periodontology and Implantology, Karnavati School of Dentistry, Karnavati University, Gandhinagar, IND

**Keywords:** male component of infertlie, india, tertiary hospital, therapeutic intervention, origins, y chromosome microdeletions, karyotyping, semen status, unproductiveness, male infertility

## Abstract

Background

Infertility and problems of impaired fecundity have been a concern through the ages and are also considerable clinical problems today, affecting many couples worldwide. Most infertility cases are primarily attributed to male factors, which play a significant role. Additionally, a substantial number of these patients exhibit suboptimal sperm parameters. The study is mainly designed for individual intervention and outcome. We aim to evaluate the demographics, etiology, utilization of treatments, and outcomes of males undergoing infertility treatment.

Methodology

We retrospectively enrolled infertile couples from January 2021 to March 2023, covering the past two years. All patients were evaluated and investigated per the study protocol to identify the cause of infertility.

Results

Two thousand three hundred forty-eight males were enrolled in the study, of whom 1,484 (63%) were found to have a standard semen analysis. A total of 868 (37%) had abnormal semen parameters. Two hundred and seventy-two (12%) patients completed the evaluation. All parameters, except for hypospermia, displayed lower percentages of motility compared to normozospermia. All semen parameters, except for hypospermia, showed a significantly lower normal morphology in comparison to normozospermia. This reduction increased by 10% for each year of age increment.

Conclusions

The study concluded by following a protocol for evaluating male patients. If an abnormal sperm parameter is identified before considering intracytoplasmic sperm injection (ICSI), it is recommended to conduct at least karyotyping and microdeletion analysis on the Y-chromosome's q arm.

## Introduction

Anomalous semen parameters noticed on semen evaluation, or imperfect sexual or ejaculatory physiological outcomes, are commonly considered male infertility features [1]. Male factors among couples seeking infertility treatment are the sole or combination with female elements [2]. The most common anomalies in semen are oligozoospermia (less than 15 million spermatozoa/mL), oligospermia (low volume, less than 1.5 mL), asthenozoospermia (less than 40% motile), and teratozoospermia (less than 4% typical forms) [3,4]. Male infertility is considered clinically severe when the concentration of sperm is less than 5 million per milliliter (severe oligozoospermia) or the inability to ejaculate the sperm (azoospermia) [5].

In 1992, there was a significant breakthrough in treating severe male infertility through the introduction of intracytoplasmic sperm injection (ICSI) in assisted reproductive technology (ART) [6]. This technology empowered many childless parents with a hope of their biological child [7]. However, this technology's newer nature always planted doubt about the growth and development of children born with this technique. The suspicion was mainly because using a single spermatozoon bypasses the processes of natural sperm selection occurring in regular fertilization [8,9]. This technique's primary concern was the potential to transmit genetic disorders to the child [10]. A significant risk of congenital abnormalities was seen in men with acute oligozoospermia or azoospermia. The threat included Y-chromosome microdeletions, cystic fibrosis gene mutations, and karyotypic chromosomal abnormalities. Chromosomal defects include Robertsonian translocation, inversion, and balanced translocation in severe oligospermia cases [11]. Microdeletion on azoospermia factor (AZF) regions of Y-chromosome sex chromosome aneuploidies like 47XXY (Klinefelter syndrome) has been reported for 1% to 3% of cases of azoospermia [12-14]. They can also have sperm that are chromosomally abnormal, even when there is no detectable genetic defect [15].

A prevalence rate of 2% to 10% for structural and chromosomal abnormalities among infertile individuals has been reported [16,17]. Among cases of severe oligospermia and nonobstructive azoospermia (NOA), the genetic abnormalities reported were 8% and 20%, respectively [18,19]. Although infertile male subjects with severe oligospermia and azoospermia may have offspring with ICSI, there remains the risk of transmission of such genetic abnormalities to these children [11].

Apart from the genetic abnormalities, the next concern is epigenetic modifications. This leads to diverse outcomes for the child's overall development [20]. These epigenetic alterations crop up due to parental characteristics related to infertility, or they can also occur due to the laboratory process involved in ICSI, which is an infertility treatment, that is required to develop embryos at the embryology laboratory of the Institute of Kidney Diseases and Research Center, Ahmedabad, Gujarat, India [21].

Problem statements 

Several studies investigated the outcomes of male factor infertility treatment, which includes drug therapy, surgery, and ART [22,23]. Many of these studies have considered only a specific factor. There is a severe lack of studies that have considered multiple factors as the reason for male factor infertility [2]. Due to the lack of data, it becomes difficult for the agencies to devise policies and strategies for addressing this issue [24]. Hence, this study will significantly help assess the role of each intervention and lacunae in the current treatment delivery systems. 

Objectives of this study

The study's objective was to investigate the causes of male factor infertility as per the semen analysis and design a protocol for evaluating male factor infertility.

## Materials and methods

We retrospectively enrolled males from the infertile couples for the past two years, from January 2021 to March 2023. All the data were extracted from the patient's case file. The Institutional Review Board of the Institute of Kidney Diseases and Research Center (IKDRC), Ahmedabad, approved this retrospective study in March 2023 (Reference no. IKDRC/ITSEC/APP/24MAR2023/07). This study was carried out following the Declaration of Helsinki. The study protocol was designed, and all the research participants were recruited and later investigated and evaluated and were explained appropriately about the objectives of the study and future scientific publication. Written informed consent was obtained before any medical or surgical interventions were conducted. All research-enrolled male patients attending the infertility clinic at IKDRC were screened for infections, including HIV, Hepatitis B surface antigen (HBsAg), Venereal Disease Research Laboratory (VDRL), and hepatitis C virus (HCV). Semen analysis was performed following the standard protocol of three days of abstinence [21].

The only case files that documented the consent of the patients for further investigation and treatment were included in the study. They underwent a standard clinical evaluation comprising a complete history, giving the physical examination details, with at least two semen analyses (according to the World Health Organization [WHO] 2015 classification) [25]. Two cases of inconsistent reports were detected that suggested abnormality (azoospermia/oligo/astheno/teratozoospermia/oligoasthenoteratozoospermia [OAT]). These cases were planned for further investigation. They were advised interventions on the base of diagnosis and were followed up for treatment.

**Figure 1:**
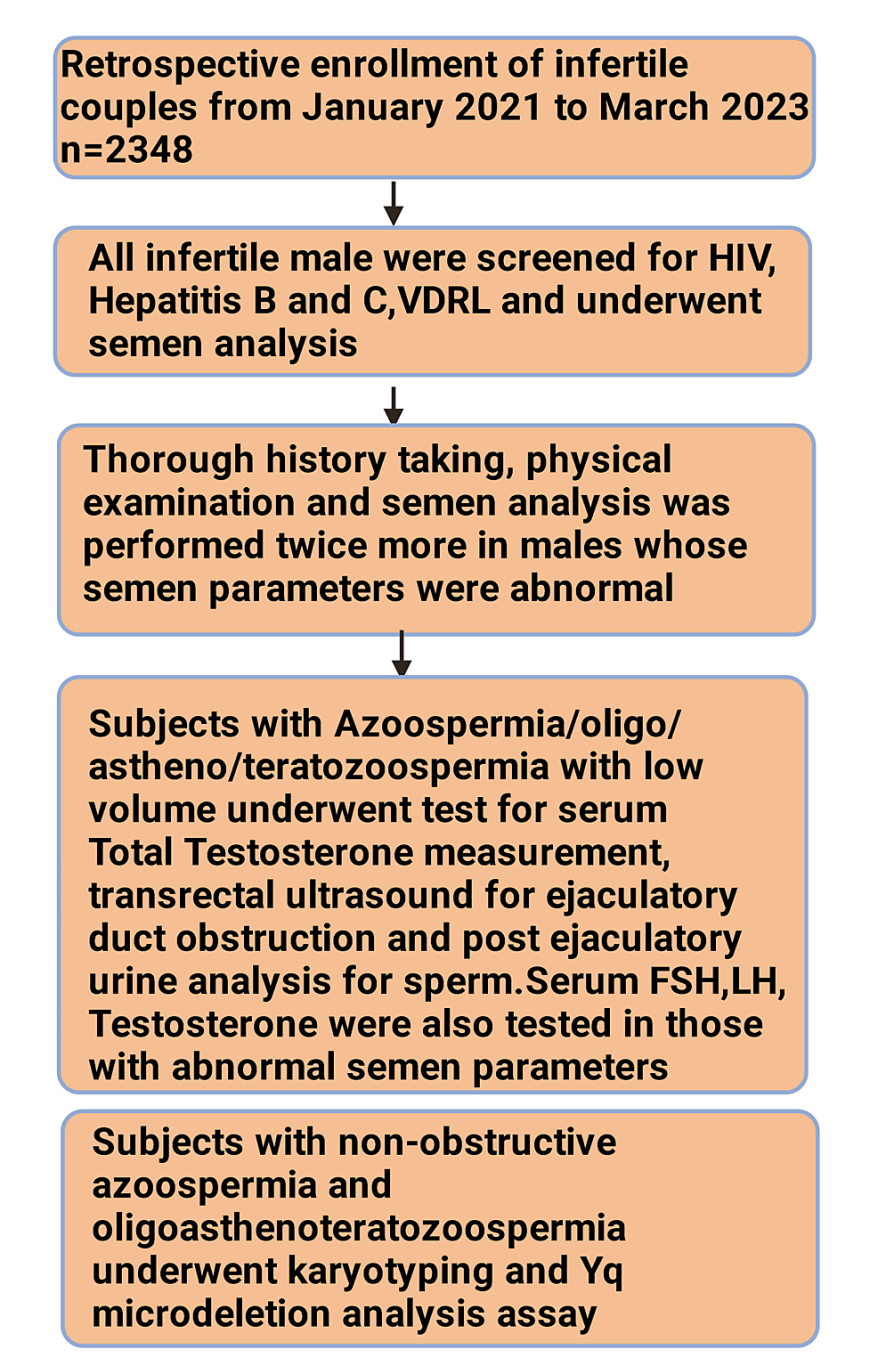
Schematic representation of the materials and methods of the study. This figure has been drawn with the premium version of BioRender (https://biorender.com/) with the license number QU25VFI0M8. Image credit: Rahnuma Ahmad. HIV, human immunodeficiency virus; VDRL, Venereal Disease Research Laboratory; FSH, follicle-stimulating hormone; LH, luteinizing hormone; Yq, Y chromosome on q arm

Supplementary investigations were done to investigate the etiology of infertility. Males with infertility factors underwent serum total testosterone measurement, transrectal ultrasound for ejaculatory duct obstruction, and post-ejaculatory urine analysis for sperms to rule out retrograde ejaculation. Serum follicle-stimulating hormone (FSH), luteinizing hormone (LH), and testosterone were tested for males with abnormal semen parameters. Patients classified as NOA and OAT underwent karyotyping and Yq (Y chromosome on the q arm) microdeletion analysis assays (Figure 1). 

Data analysis was conducted using STATA-15 (STATA is a syllabic abbreviation of the words statistics and data; StataCorp. LLC, College Station, TX, USA), with a significance level set at a *P*-value of <0.05. Descriptive analysis was used to elucidate the characteristics of all exposures and outcomes. To examine the connections between exposures (semen parameters) and outcomes (volume, sperm concentration, motility, mobility, and normal morphology), we used a multiple regression model. In this regression model, age served as a covariate, and significance within the model was established when *P *< 0.1.

## Results

**Figure 2:**
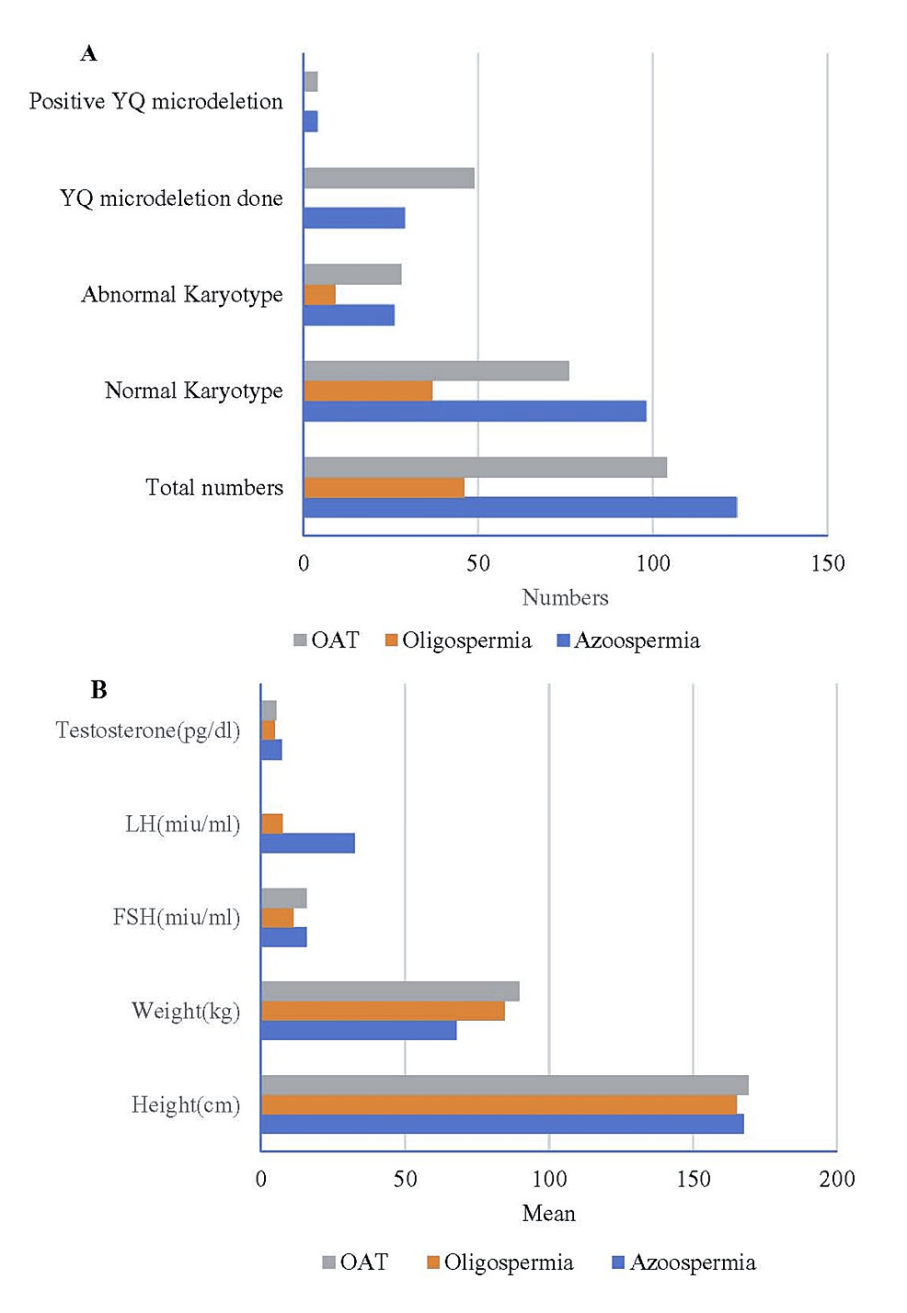
Results of chromosomal and hormonal investigation: data presented as (A) number and (B) mean.

Two thousand three hundred forty-eight males were enrolled in the study, of whom 1,484 (63%) were found to have normozoospermia. OATs was found in 11% (259), azoospermia in 7.5% (178), hypospermia in 7.4% (174), oligozoospermia in 6.6% (156), asthenozoospermia in 2.6% (62), hyperspermia in 1.1% (27), giobozoospermia in 0.8% (2) teratozoospermia in 0.8% (2), hypooligospermia in 0.8% (2), hyperoligospermia in 0.4% (1), cryptozoospermia in 0.4% (1) (Table 1). The mean (standard deviation [SD]) age was 34.96 years. Out of 178 azoospermia patients, only 124 (69.6%) opted for further evaluation, and karyotyping was done. Ninety-eight (79%) patients had a normal karyotype, while 26 (21%) had an abnormal karyotype. Yq microdeletion testing was conducted for 29 patients, and among them, four (12.7%) had Y microdeletion, along with elevated levels of FSH (15 miu/mL), LH (32 miu/mL), and testosterone (7.17 pg/dL) in cases of OATs. Out of 259 OAT patients, only 104 (40.1%) patients opted for further investigation. Among the further investigated patients, 76 (72.1%) patients had a normal karyotype and 28 (26.9%) patients had an abnormal karyotype. Forty-nine (18.01%) patients were further investigated for Yq microdeletion, of which 4 (8%) were positive with FSH levels of 15.8 miu/mL, LH levels of 9.3 miu/mL, and testosterone levels of 5.42 pg/dL (Figure 2).

**Table 1:** Demographic data of semen analysis. Out of 2,348 cases, 862 (36.7%) were found to have abnormal sperm. ^*^Data presented as number with the percentage in the parenthesis. ^ɤ^Data presented as mean ± SD. SD, standard deviation

Semen parameter	Number (%)^*^	Age (Years)^ɤ^	Viscosity (PI)	Volume (mL)^ ɤ^	Sperm concentration (million per mL)^ɤ^	Motility (%)^ɤ^	Mobility (%)^ɤ^	Normal morphology (%)^ɤ^
Normozospermia	1,484 (63)	34.0 ± 5.7	1	2.4 ± 2.5	72 ± 25.5	41.3 ± 7.1	23 ± 13	6.7 ± 1.91
OAT	259 (11)	33.8 ± 6.27	1	2.35 ± 1.38	3.58 ± 3.34	13 ± 7.0	12 ± 4.9	2.8 ± 0.69
Azoospermia	178 (7.5)	33.2 ± 6.53	1	1.82 ± 1.47	0	00	00	0
Hypospermia	174 (7.4)	35.0 ± 7.07	1	1.0 ± 1.04	66 ± 25.9	42 ± 7.74	19 ± 8.9	6.4 ± 3.5
Oligozoospermia	156 (6.6)	33.8 ± 8.5	1	2.17 ± 1.13	6.6 ± 4.70	29.7 ± 9.4	14 ± 6.9	4.8 ± 1.63
Asthenozoospermia	62 (2.6)	32.5 ± 8.64	1	2.90 ± 4.97	40.2 ± 21.8	11.7 ± 9.2	10 ± 6.9	4.2 ± 1.5
Hyperspermia	27 (1.1)	34 ± 4.85	3	6.15 ± 1.04	66.6 ± 25.7	42 ± 7.7	21 ± 6.1	6.74 ± 2.30
Globozospermia	2 (0.8)	38.0 ± 8.48	1	2.50 ± 0.35	26.4 ± 36.0	16 ± 22.6	14 ± 20	0
Teratozospermia	2 (0.8)	27.5 ± 4.95	1	2.22 ± 1.13	48.0 ± 11.3	36 ± 5.7	11 ± 2.1	0
Hypo + oligo	2 (0.8)	33.0 ± 15.6	1	0.65 ± 0.49	6.5 ± 4.95	24 ± 12	12 ± 0	3.5 ± 0.71
Hyper + oligo	1 (0.4)	30	1	5.2	11	35	7	3.5
Cryptozospermia	1 (0.4)	38	1	0.5	1	0	0	0

**Figure 3:**
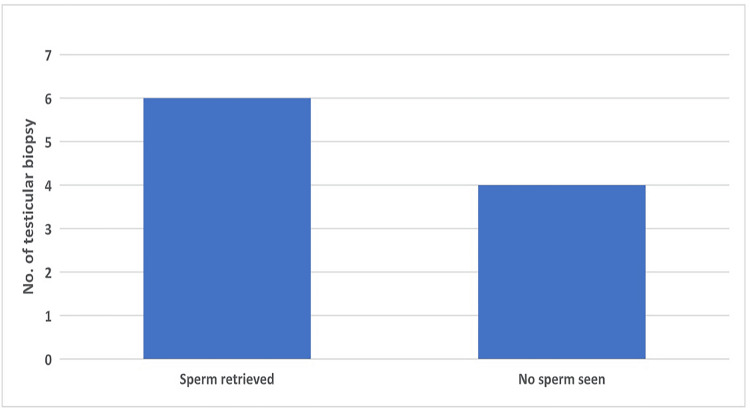
Results of testicular biopsies. Bars represent numbers.

Oligospermia was screened in 156 patients, of which only 46 (29.4%) patients opted for further treatment. Among those, 37 (80.5%) patients had normal karyotyping and 9 (19.56%) had abnormal karyotyping with FSH levels of 11.2 miu/mL, LH levels of 7.4 miu/mL, and testosterone level of 4.6 pg/dL (Table 2). All 274 patients with azoospermia, oligospermia, and OATs underwent ultrasound as part of the workup. Among those, varicocele was detected in six (4.83%) azoospermia patients, two (4.34%) oligospermia patients, and one (0.96%) OAT patient. One undescended testis was seen in the azoospermia patient, 12 abnormal large-size testes in the azoospermia patient, and two abnormal sizes and two small-size testes were detected in the oligospermic patient (Table 3). All OAT and oligospermia patients were advised for in vitro fertilization and ICSI protocol. Ten azoospermia patients opted for testicular biopsy (Table 4). Sperms were retrieved in six patients, whereas sperms were absent in the other four. Post ICSI, 17 embryos and 10 blastocysts (Table 4) were formed (Figure 3). 

**Table 2:** Chromosomal and hormonal investigations. *Data presented as number with the percentage in the parenthesis. ^ɤ^Data presented as mean ± SD. FSH, follicle-stimulating hormone; LH, luteinizing hormone; SD, standard deviation

Types	Total numbers (%)^*^	Normal karyotype (%)^*^	Abnormal karyotype (%)^*^	Yq microdeletion done (%)^*^	Positive Yq microdeletion (%)^*^	Height (cm)^ɤ^	Weight (kg)^ ɤ^	FSH (miu/mL)^ɤ^	LH (miu/mL)^ɤ^	Testosterone (pg/dL)^ɤ^
Azoospermia	124 (79.48)	98 (79.03)	26 (20.96)	29 (20.96)	4 (3.22)	167.4 ± 10.2	67.89 ± 11.9	15.9 ± 13.1	32.5 ± 16.2	7.17 ± 8.2
Oligospermia	46 (29.4)	37 (80.5)	9 (19.56)	Not done	Not done	165.1 ± 12.5	84.5 ± 12.7	11.2 ± 6.7	7.4 ± 4.1	4.65 ± 1.64
OAT	104 (66.6)	76 (73.07)	28 (26.92)	49 (47.11)	4 (3.84)	169.1 ± 9.7	89.6 ± 12.5	15.8 ± 6.6	9.3 ± 3.5	5.42 ± 3.46

**Table 3:** Ultrasonography findings. ^*^Data presented as number with the percentage in the parenthesis. OAT, oligoasthenoteratozoospermia

	Varicocele, *n *(%)	Undescended testes, *n *(%)	Abnormal size testes, *n *(%)	Small testes, *n *(%)
Azoospermia	6 (4.83)	1 (0.80)	12 (9.67)	Nil
Oligospermia	2 (4.34)	Nil	2 (4.34)	2 (4.34)
OAT	1 (0.96)	Nil	Nil	Nil

**Table 4:** Outcome of testicular biopsies. ^*^Data are presented as the number with the percentage in the parenthesis.

	Number of testicular biopsies (%)	Number of outcomes of testicular biopsy (%)		Number of embryo formed (%)
		Sperm retrieved	No sperm	
Azoospermia	10 (8.06)	6 (60)	4 (40)	17 (13.70%) embryo + 10 blastocysts (8.06%)

In the multiple regression analysis, the results indicate that oligozoospermia, OAT, and hypospermia exhibited higher semen volume compared to normozospermia by 0.26 mL (95% confidence interval [CI] = 0.05-0.46; *P *= 0.016), 0.41 mL (95% CI = 0.24-0.58, *P *< 0.001), and 0.20 mL (95% CI = 0.004-0.40), respectively. Conversely, other parameters showed lower semen volume compared to normozospermia. Additionally, each year of age increment was associated with a significant increase of 0.01 mL in semen volume (95% CI = 0.002-0.02; *P *= 0.013) (Table 5).

**Table 5:** Association of semen parameters with volume, sperm concentration, motility, mobility, and normal morphology. A multiple regression model was used to estimate the *P*-value, and the regression model was adjusted by the age of the participants. A *P*-value of <0.05 is considered significant, whereas for the covariate, *P *< 0.10 is considered as potential effect in the regression model. ^*^Others (globozoospermia, cryptozoospermia, teratozoospermia, hypo + oligo, hyper + oligo, hyperspermia).

Semen parameters	Volume (mL)		Sperm concentration (millions/mL)		Motility (%)		Mobility (%)		Normal morphology	
	β-(95% CI)	P	β-(95% CI)	P	β-(95% CI)	P	β-(95% CI)	P	β-(95% CI)	P
Normozospermia	Ref.		Ref.		Ref.		Ref.		Ref.	
Oligozoospermia	0.26 (0.05-0.46)	0.016	-65 (-69 to -61)	<0.001	-11.8 (-13 to -11)	<0.001	-9.10 (-10.9 to -7.27)	<0.001	-2.22 (-2.54 to -1.90)	<0.001
Asthenozooepermia	0.24 (-0.09 to 0.56)	0.156	-33 (-38 to -27)	<0.001	-30 (-32 to -28)	<0.001	-14 (-16 to -11)	<0.001	-2.64 (-3.14 to -2.15)	<0.001
OAT	0.41 (0.24 to 0.58)	<0.001	-68 (-71 to -65)	<0.001	-29 (-30 to -28)	<0.001	-12 (-13 to -10)	<0.001	-3.97 (-4.23 to -3.72)	<0.001
Azoospermia	0.14 (-0.06 to 0.34)	0.174	-71 (-75 to -68)	<0.001	-41 (-43 to -40)	<0.001	-23 (-25 to -22)	<0.001	-6.72 (-7.02 to -6.42)	<0.001
Hypospermia	0.20 (0.004 to 0.40)	0.046	-7.13 (-10.6 to -3.64)	<0.001	0.43 (-0.71 to 1.56)	0.461	-4.43 (-6.15 to -2.72)	<0.001	-0.21 (-0.51 to 0.09)	0.165
Others^*^	-0.19 (-0.62 to 0.23)	0.368	-15.5 (-23 to -8.10)	<0.001	-3.89 (6.31 to -1.48)	0.002	-4.52 (-8.18 to -0.87)	0.015	-1.05 (-1.70 to -0.40)	0.002
Age (years)	0.01 (0.002 to 0.02)	0.013	0.13 (-0.02 to 0.28)	0.089	-3.89 (-0.07 to -1.48)	0.433	-0.01 (-0.08 to 0.06)	0.798	0.01 (-0.002 to 0.02)	0.085

Regarding sperm concentration, all semen parameters exhibited significantly lower concentration (*P *< 0.001) compared to normozospermia, with age exerting a significant influence on the model. Specifically, each year of age increase was associated with a higher concentration of 0.13 million/mL. For sperm motility, all parameters, except for hypospermia, displayed lower percentages of motility compared to normozospermia. Age did not have a significant impact on this model. Similarly, all other semen parameters demonstrated lower mobility percentages, with age showing no influence on the regression model. With respect to normal morphology, except for hypospermia, all other semen parameters also exhibited significantly lower normal morphology compared to normozospermia, with the reduction increasing by 10% for each year of age increment.

## Discussion

Our study highlights the real-time scenario in tertiary in vitro fertilization (IVF) centers. Most of the patients came for the first time for a semen report. Our study found that the standard semen parameter was seen in 63% (1,484), abnormal semen parameter was seen in 37% (868), and OATs in 11% (259) of cases, with azoospermia detected in 7.5% (178) of cases. Mahmud et al. conducted another study on male infertility and reported normozoospermia in 60%, 85%, and 83% in Bangladesh, India, and Pakistan, respectively. OAT cases were 13.1% in Bangladesh, 8% in India, and 16.8% in Pakistan. Azoospermia cases were 15.5% in Bangladesh, 8% in India, and 6.6% in Pakistan [26].

Most patients with abnormal semen parameters were advised for the ART option. Counseling of the couple was conducted, and they were recommended for further investigation to determine the cause of abnormal semen parameters. Despite counseling, out of 862 patients, 274 (31.7%) opted for further management. A study by Kaushal et al. stated that despite the three-year duration of our research, among the 422, only 103 (25%) patients completed the assigned treatment, while another 134 were awaiting initiation of therapy [27]. Out of abnormal 862 patients, OATs were in 259 (30%) patients, azoospermia in 178 (20.6%), hypospermia in 174 (20%), oligozoospermia in 156 (18%), and asthenospermia in 62 (7%). In a study by Umashankar et al., the majority (32%) of patients had OAT, followed by azoospermia (27%), OA (26%), and oligospermia (8%) [28]. For further evaluation, patients were advised for an ultrasound of the testis, estimation of hormonal parameters like FSH, testosterone, and karyotyping. Patients with normal karyotyping were advised for Yq microdeletion. Before proceeding with ICSI, a genetic analysis of all the patients with abnormal semen parameters was done. In our study, with azoospermia patients, out of 124(79.48%), 26 (20.96%) patients had abnormal karyotyping. Yq microdeletion was carried out in 29 (23.28%) patients, out of which four (13.79%) patients were detected positive. Out of 259 (11%) cases of oligoasthenospermia, 104 (40.15%) patients had had karyotyping done. The results showed that 28 (10.81%) had abnormal karyotypes. Forty-nine (18.91%) patients were investigated for microdeletion, which showed four (8.16%) positive patients. Out of 46 (29.4%) patients with oligospermia, nine (19.56%) had abnormal karyotypes.

The patients who demonstrated abnormal karyotypes were counseled regarding the chances of abortion. There is an absolute lack of any relationship between genotype and testicular histology, which leads to the absence of any clinical parameters by which microdeletions of infertile males can be predicted [29]. The most effective therapeutic option for an infertile population is to extract spermatozoa from the testes of the male suffering from NOA followed by ICSI [30]. Few males in these categories may contain Y-chromosome microdeletions, which can contribute to a pregnancy predicted based on currently used routine investigations. It becomes essential for males in such a category to undergo screening for chromosomal deletions. This is not just essential for the diagnosis of the etiology of infertility but also the counseling of ART. The phenotypes associated with chromosomal microdeletions in the azoospermia factor (AZF) zones differ [31,32].

**Figure 4:**
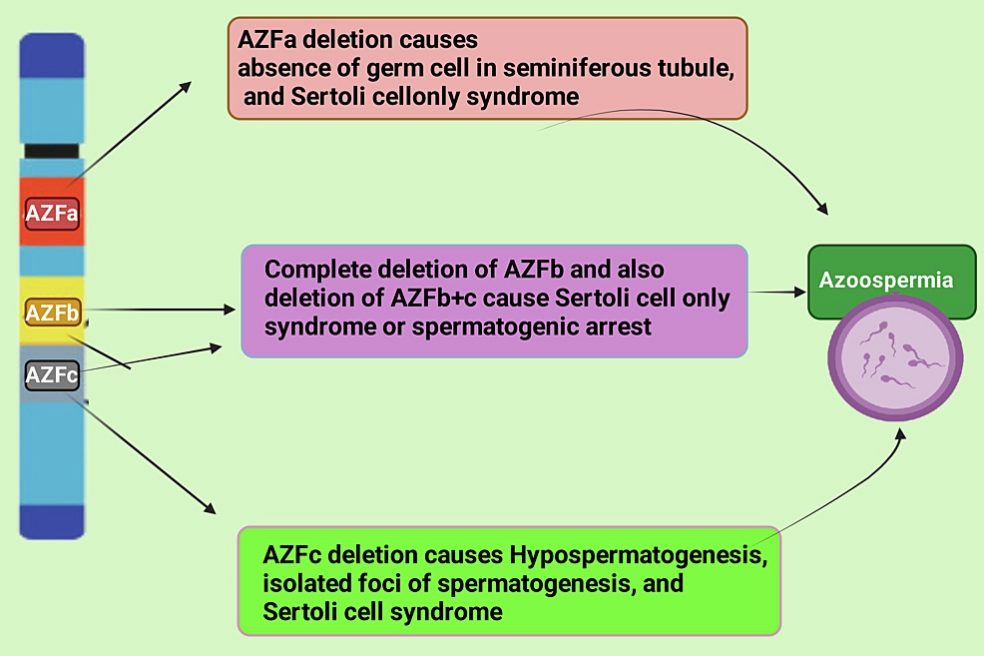
Different male reproductive phenotypes associated with the deletion of AZF. This figure has been drawn with the premium version of BioRender (https://biorender.com/) with the license number KZ25RFMF1M. Image credit: Rahnuma Ahmad. AZF, azoospermia factor

Azoospermia results from the complete chromosomal deletions of AZFc and AZF B plus factor C (AZFb+c). These are characterized by Sertoli cells seen in the histologic picture [32]. Chromosomal deletions in the AZFa region have been linked with a total absence of germ cells in the seminiferous tubules and del Castillo syndrome [33,34]. Moreover, the deletion of the AZFc region is associated with a wide range of phenotypes, ranging from hypospermatogenesis-isolated foci of spermatogenesis and Sertoli cells syndrome [35] (Figure 4).

In their research study, Machev et al. showed that partial deletion of AZFc or gene conversion frequently causes spermatogenic failure [36]. A probable justification for the inconsistent phenotype is the progressive regression of germinal epithelium over time, which has been reported in patients with AZFc deletions [37]. The omission of AZFc, followed by AZFb and AZFa, from all three AZF regions, is the most frequent [38].

**Figure 5:**
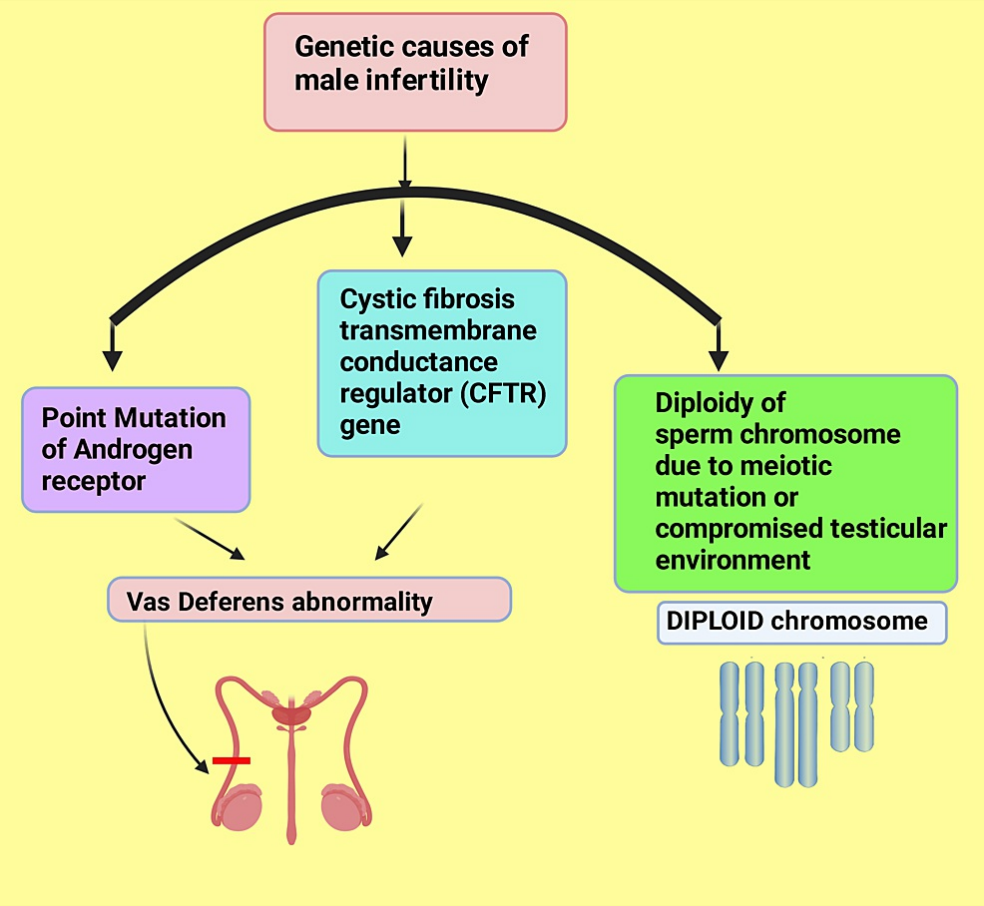
Chromosomal abnormalities linked to male infertility. This figure has been drawn with the premium version of BioRender (https://biorender.com/) with the license number BK25VFHIB2. Image credit: Rahnuma Ahmad.

A primary chromosomal study is essential to rule out duplication, deletion, and microdeletion. Most frequently, chromosomal abnormalities often lead to the chromosomal disorder of male infertility. A well-established cause of male infertility is the Klinefelter syndrome (XXY) and specific translocations [37]. Two critical genes associated with spermatogenic failure are mutation in the androgen receptor and the cystic fibrosis transmembrane conductance regulator (CFTR) gene. These are primarily related to congenital Sertoli cell-only syndrome [38]. Diploidy, originating from either meiotic mutations or a compromised testicular environment, is the most common sperm chromosome anomaly (Figure 5).

For patients with azoospermia, ultrasound findings suggested one patient had an undescended testis, while 12 others had abnormal-sized testes. Small testes were detected in two patients, and varicocele was detected in one patient with OATs. Imaging is essential for differentiating the causes of obstructive azoospermia, which may be correctable and related to infertility, from the causes of NOA. It is also critical to rule out life-threatening conditions associated with infertility and genetic disorders that can be transmitted to offspring [39].

After the complete workup discussion of percutaneous epididymal sperm aspiration (PESA)/testicular sperm aspiration (TESA) with the patients, prior consent was taken for donor sperm or oocyte freezing if sperms were not found in the biopsy. If sperms were not detected in the biopsy, the tissue was sent for diagnostic histopathology. Ten azoospermia patients opted for testicular biopsy. Sperms were retrieved from six (60%) patients, and no sperms were detected in the other four (40%) patients. Therapeutic and diagnostic biopsy was preferred after oocyte retrieval.

In the multiple regression analysis, the results indicate that oligozoospermia, OAT, and hypospermia exhibited higher semen volume compared to normozoospermia among male infertility cases. Our findings were in similar line with earlier studies [40,41]. This study also detected the aging process has a substantial influence on sperm quantity and quality, similar to previous research reports [42,43].

Limitations of this study

The study is conducted in a specific population strata, so there can be a potential referral bias. The subjects who reported to our hospital were primarily from lower socioeconomic strata and with low levels of education. Subjects from higher income and educational groups should have been included to obtain better research results. Moreover, confounding factors such as patients from high and middle socioeconomic strata and with suitable educational qualifications were very few in the cohort - the social belief of the area where the patient reported. Counseling by different clinicians also affected the study. These issues have the probability of influencing our study results.

## Conclusions

The study aims to establish a protocol for evaluating male patients and providing counseling regarding the importance of further investigation in cases of abnormal sperm. Additionally, it seeks to discuss the advantages and disadvantages of testicular biopsy and ICSI before presenting these options to infertile couples. This study suggests a productive and therapeutic approach in oligospermia, OAT, and azoospermia after sperm retrieval. Considering the difficulty in predicting the presence of microdeletions based on current technology, it becomes essential for all infertile males to undergo screening before using ART. If a patient presents with symptoms suggestive of cystic fibrosis and obstructive azoospermia with a CFTR gene mutation or any other genetic abnormality, it is essential to counsel the couple about the potential risk of disease transmission. Additionally, when we conducted the multiple regression analysis, the results indicated that oligozoospermia, OAT, and hypospermia exhibited higher semen volume compared to normozospermia by 0.26 mL (95% CI = 0.05-0.46; *P *= 0.016), 0.41 mL (95% CI = 0.24-0.58; *P *< 0.001), and 0.20 mL (95% CI = 0.004-0.40), respectively. Conversely, other parameters showed lower semen volume compared to normozospermia. Additionally, each year of age increment was associated with a significant increase of 0.01 mL in semen volume (95% CI = 0.002-0.02; *P *= 0.013).
